# Physico-Chemical and Microbial Analysis of Selected Borehole Water in Mahikeng, South Africa

**DOI:** 10.3390/ijerph120808619

**Published:** 2015-07-23

**Authors:** Lobina Palamuleni, Mercy Akoth

**Affiliations:** Department of Geography and Environmental Sciences, Mafikeng Campus, North West University, Private bag X2046, Mmabatho 2735, South Africa

**Keywords:** drinking water, *Escherichia coli*, physico-chemical, water quality

## Abstract

Groundwater is generally considered a “safe source” of drinking water because it is abstracted with low microbial load with little need for treatment before drinking. However, groundwater resources are commonly vulnerable to pollution, which may degrade their quality. An assessment of microbial and physicochemical qualities of borehole water in the rural environs of Mahikeng town, South Africa, was carried out. The study aimed at determining levels of physicochemical (temperature, pH, turbidity and nitrate) and bacteriological (both faecal and total coliform bacteria) contaminants in drinking water using standard microbiology methods. Furthermore, identities of isolates were determined using the API 20E assay. Results were compared with World Health Organisation (WHO) and Department of Water Affairs (DWAF-SA) water quality drinking standards. All analyses for physicochemical parameters were within acceptable limits except for turbidity while microbial loads during spring were higher than the WHO and DWAF thresholds. The detection of *Escherichia coli*, *Salmonella* and *Klebsiella* species in borehole water that was intended for human consumption suggests that water from these sources may pose severe health risks to consumers and is unsuitable for direct human consumption without treatment. The study recommends mobilisation of onsite treatment interventions to protect the households from further possible consequences of using the water.

## 1. Introduction

On a global scale, groundwater represents the world’s largest and most important source of fresh potable water [1]. Groundwater provides potable water to an estimated 1.5 billion people worldwide daily [2] and has proved to be the most reliable resource for meeting rural water demand in the sub-Saharan Africa [3,4]. Due to inability of governments to meet the ever-increasing water demand, most people in rural areas resort to groundwater sources such as boreholes as an alternative water resource. Thus, humans can abstract groundwater through a borehole, which is drilled into the aquifer for industrial, agricultural and domestic use. However, groundwater resources are commonly vulnerable to pollution, which may degrade their quality.

Generally, groundwater quality varies from place to place, sometimes depending on seasonal changes [5,6], the types of soils, rocks and surfaces through which it moves [7,8]. Naturally occurring contaminants are present in the rocks and sediments. As groundwater flows through the sediments, metals such as iron and manganese are dissolved and may later be found in high concentrations in the water [9]. In addition, human activities can alter the natural composition of groundwater through the disposal or dissemination of chemicals and microbial matter on the land surface and into soils, or through injection of wastes directly into groundwater. Industrial discharges [10], urban activities, agriculture [9], groundwater plumage and disposal of waste [11] can affect groundwater quality. Pesticides and fertilizers applied to lawns and crops can accumulate and migrate to the water tables thus affecting both the physical, chemical and microbial quality of water.

In rural Africa, where the most common type of sanitation is the pit latrines, this poses a great risk on the microbial quality of groundwater. For instance, a septic tank can introduce bacteria to water and pesticides and fertilizers that seep into farmed soils can eventually end up in the water drawn from a borehole. Poor sanitary completion of boreholes may lead to contamination of groundwater. Proximity of some boreholes to solid waste dumpsites and animal droppings being littered around them [11] could also contaminate the quality of groundwater. Therefore, groundwater quality monitoring and testing is of paramount importance both in the developed and developing world [12]. The key to sustainable water resources is to ensure that the quality of water resources are suitable for their intended uses, while at the same time allowing them to be used and developed to a certain extent.

Although surface water is the main source of water supply in South Africa, ground water is extensively utilized, particularly in rural and arid areas with only about half of the country’s groundwater resources (around 7500 million m^3^/a) being used [13]. Due to South Africa’s unpredictable rainfall, high evaporation rates and low conversion of rainfall to runoff, South Africa is a water stressed country, where demand is fast approaching available supply [14]. This, coupled with rising water consumption, is placing increasing demands on the nation’s existing water resources. The North West Province, being generally an arid province has all of these water resource constraints.

Mahikeng, the capital city of the North West Province is one of several towns in South Africa whose residents especially in rural areas depend on groundwater resources. Groundwater is piped to the Mahikeng Water Treatment Plant where the flows are combined and the water is chlorinated. From the treatment plant the water is reticulated into the town. However, the majority of the surrounding peri-urban areas are not connected to the water system and individuals in these communities make use of boreholes, which are either equipped with various electric, diesel or wind pumps. The quality of water from these sources is variable, but usually in some areas it may contain huge amounts of nitrates and this is of particular concern [13]. High levels of salinity, high hardness and microbiological problems have also been reported in groundwater. Water quality problems have partly been associated with inadequate sanitation [13].

The common sanitation system applicable in most of the peri-urban areas of Mahikeng is Ventilated Improved Pit latrines (VIP) and/or dug pits which are dry systems and do not use water. However, it has been observed that the sources of water in some villages are in close proximity to human settlements and thus there is an inherent risk of pollution of the groundwater aquifer which supplies the village. It is therefore against this background that the physicochemical and bacteriological parameters of borehole water in Mahikeng were assessed to ascertain whether the borehole water was within the acceptable standards for human consumption as set by the World Health Organisation.

## 2. Experimental Section

### 2.1. Study Area

**Figure 1:**
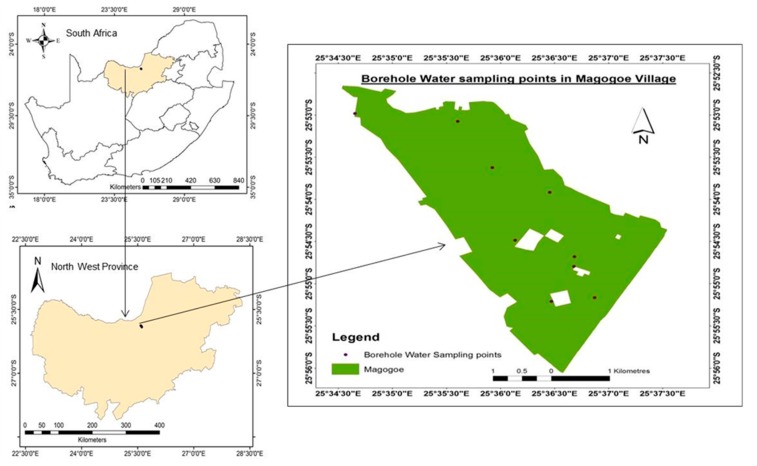
Map of the study area within South Africa and sampling points.

The study was conducted in Mahikeng, North West Province of South Africa in a village called Magogoe, about 8 km from Mahikeng town (Figure 1). Mahikeng is located within Latitude −25° 51′ S and Longitude 25° 38′ E, covering a total area of 24.57 km^2^. Magogoe village is located within Latitude −25° 53′ 12.99″ and Longitude 25° 36′ 39.98″, and covers an area of 3.75 km^2^.

### 2.2. Collection of Water Samples

Drinking water samples (24) from eight randomly selected boreholes in the Magogoe village were collected for microbiological and physico-chemical analyses. Water samples were collected twice in June, when the temperatures were lower and September, when the temperatures were warmer, in order to establish the seasonal variations on physico-chemical and bacteriological parameters present in the water. Water samples were collected from the selected boreholes using three sterile 250 mL plastic bottle for each sample.

### 2.3. Physico-Chemical Analyses

#### Determination of Temperature, pH, Turbidity, and Nitrate Levels in Drinking Water Samples

The pH of water samples was analysed on-site using a pH meter (Model 300408.1, Denver Instruments Company, Bohemia, New York, USA) which was previously calibrated using two buffer solutions, pH 4 and pH 7. Thermometer reading in °C was used to record the temperature of the water samples while water turbidity was determined using a portable turbidimeter (TB200-IR-10). The concentrations of nitrate were determined in the laboratory using UV/Vis spectrophotometer at 410 nm using EDTA as described by American Public Health Association [15].

### 2.4. Microbiological Analyses of Water Samples

Water samples were analysed immediately after collection, for the presence of total coliforms and E. coli (bacterial indicator for faecal contamination) using membrane filtration method [16]. Aliquots of 50 mL from each samples was filtered using 0.45 µm paper filters. The filters were placed on mFC and mENDO agar and plates were incubated aerobically at 45 °C and 37 °C respectively for 24 h. Blue and metallic sheen colonies on MFc and mENDO agar plates were purified and used for bacteria identification tests. The isolates were subjected to both preliminary Gram staining [17]; oxidase, citrate utilization [18]; Triple Sugar Iron tests [19,20] and confirmatory biochemical identification tests (EnteroPluri-Test, Ref: 78618-78619) to screen for characters of bacteria belonging to the family *Enterobacteriaceae*.

### 2.5. Data Analysis

Data for microbial and physico-chemical contaminants in drinking water samples were recorded and analysed for total coliforms, *E. coli*, pH, turbidity, and nitrate. Mean and standard deviations were calculated from the results of the analysis of the three samples per sampling point. Water quality results were compared with the Department of Water Affairs and the World Health Organisation drinking water standards.

## 3. Results and Discussion

### 3.1. Physico-Chemical Analyses

Table 1 shows the physico-chemical analyses determined in winter and spring. Generally, most of the physico-chemical parameters in the majority of the boreholes were within the DWAF and WHO water standards for domestic use. On the contrary, the turbidity and nitrate concentrations of water from some of the boreholes were above the required limits. When the results from winter and spring are compared, it is evident that the temperature between the two seasons was relatively constant. This might have resulted from the fact that these boreholes were within the same area and protected from temperature variations.

**Table 1:** Physico-chemical analyses of water samples for winter and spring.

	Temperature (°C)		pH		Turbidity (NTU)		Nitrate (mg/L)	
	Winter	Spring	Winter	Spring	Winter	Spring	Winter	Spring
Borehole 1	15.3 ± 3.6	20.4 ± 0.6	7.7 ± 0.1	7.4 ± 0.03	0.9 ± 0.4	0.6 ± 0.1	6.7 ± 2.7	6.4 ± 3.1
Borehole 2	17.6 ± 0.3	21.2 ± 2.0	7.4 ± 0.09	7.4 ± 0.02	1.5 ± 0.7	0.8 ± 0.1	4.1 ± 0.1	3.2 ± 0.4
Borehole 3	23.1 ± 0.2	20.3 ± 4.4	7.3 ± 0.02	7.3 ± 0.05	**40.9 ± 3.9**	**37.7 ± 0.6**	3.2 ± 0.2	2.0 ± 1.5
Borehole 4	19.5 ± 1.6	21.8 ± 1.4	7.6 ± 0.04	7.7 ± 0.02	1.2 ± 0.5	0.8 ± 0.1	7.6 ± 2.2	4.4 ± 2.2
Borehole 5	25.1 ± 0.1	22.2 ± 1.4	7.6 ± 0.03	7.8 ± 0.01	0.6 ± 0.1	0.7 ± 0.1	4.2 ± 1.2	2.7 ± 2.0
Borehole 6	22.7 ± 0.4	23.2 ± 2.7	7.2 ± 0.04	7.4 ± 0.03	0.5 ± 0.2	1.2 ± 0.5	**17.1 ± 4.4**	**11.1 ± 7.2**
Borehole 7	22.4 ± 0.2	23.9 ± 0.3	7.3 ± 0.02	7.3 ± 0.03	**31.1 ± 11.8**	**15.9 ± 6.9**	3.6 ± 3.7	3.7 ± 0.5
Borehole 8	20.9 ± 0.9	—	7.4 ± 0.03	_—_	1.3 ± 0.4	_—_	1.8 ± 0.4	_—_
DWAF	No standards		≥5 to ≤9.7		≤1 NTU		<11 mg/L	
WHO	No standards		≥7 to ≤9.2		5 NTU		50 mg/L	

Temperature is one of the most important ecological and physical factor which has a profound influence on both the living and non-living components of the environment, thereby affecting organisms and the functioning of an ecosystem. Although temperature generally influences the overall quality of water (physico-chemical and biological characteristics), there are no guideline values recommended for drinking water. Therefore, having analysed temperature for the collected borehole water samples during winter and spring, the overall mean values were 20.8 °C and 22.9 °C, respectively (Table 1).

The pH of water is important because many biological activities can occur only within a narrow range, thus any variations beyond an acceptable limit could be fatal to a particular organism [5]. In the present study, all borehole water samples collected during both seasons had pH values within the recommended ranges for both DWAF and WHO drinking water standards. The values ranged from 7.2 to 7.8 for both seasons. Therefore, the pH of the borehole water in the study area could be classified as suitable for drinking purposes.

Turbidity is defined as the measure of the clarity or cloudiness of water and the values are attained by measuring the scattering and absorbing effect that suspended particles have on light [21]. Turbidity values ranged from 0.5 NTU to 40.9 NTU for all the water samples. Turbidity results for the other boreholes during both seasons were within WHO standards except for boreholes 3 and 7. The plausible explanation for high turbidity from borehole 3 and 7 could be the use of a hand pump resulting from corrosion. Corrosion may cause permeability of the hand pump such that soil particles seep into the water thereby causing high turbidity levels [22].

Nitrate concentration in most of the boreholes is below both DWAF and WHO guidelines (Table 1). Nitrate levels therefore did not appear to be a serious water quality problem except for borehole 6 whose nitrate concentration during both seasons was above the permissible DWAF standards though the values were within WHO standards. This could have been due to this borehole being located in close proximity to an animal shelter thereby causing surface pollution. Oxidation of ammonia form of nitrogen from animal and human wastes to nitrite is a possible way of nitrate entry into the groundwater aquifer [23]. In higher concentrations, nitrate may produce a disease known as Methemoglobinemia (blue baby syndrome) which generally affects bottle-fed infants. Repeated doses of nitrates on ingestion may also cause carcinogenic diseases [24].

### 3.2. Bacteriological Analyses

Total coliform bacteria are known as “indicator organisms” meaning that their presence provides indication that other disease causing organisms may also be present in the water body. The total bacterial count in the borehole water sampled during winter ranged from <1 to 44.1 cfu/100 mL. However, during spring higher values were recorded (1.0 to 579.4 cfu/100 mL) (Table 2). It can be noted that except for borehole 7, all the water samples from the other boreholes were within the permissible standards of DWAF and WHO drinking water standards. However, five of the borehole water samples collected during spring did not conform to the set guidelines for drinking water.

**Table 2:** Results of the bacteriological analyses.

	Total Coliform Bacteria (cfu/100 mL)	
	Winter	Spring
Borehole 1	<1	133.3
Borehole 2	<1	272.3
Borehole 3	1.0	5.2
Borehole 4	2.0	1.0
Borehole 5	<1	579.4
Borehole 6	<1	172.2
Borehole 7	**44.1**	461.1
Borehole 8	1.0	_—_
DWAF	≤10 cfu/100 mL	
WHO	≤10 cfu/100 mL	

The high total coliform count during spring could be attributed to the increase in temperature. Temperature affects the rate of proliferation of micro-organisms [11]. Another possible cause of high coliform count could be the proximity of certain boreholes to pit latrines and poor sanitary completion of boreholes may have led to contamination of groundwater. Total coliforms can also originate from environmental sources such as soils or from biofilms.

Although information on the depth of the sampled boreholes was not available, another possible cause of microbial contamination is the depth of the borehole [7,9]. Minimum depth of a borehole is 40 m such that microbial contamination from surface sources is removed within the first 30 m as groundwater passes through saturated sand and non-fissured rock. In unsaturated zone, no more than 3 m may be necessary to purify groundwater. However, in fractured aquifer, microbial contaminants can rapidly pass through the unsaturated zone to the water table [9].

During the study, it was observed that some of the boreholes are electrical such that the water is pumped into pipes for distribution. Rusty pipes affect the quality of water by allowing seepage of microbial contaminants into the borehole [22].

#### Selective Detection of Faecal and Total Coliform Bacteria

All the 21 presumptive isolates from m-FC agar were subjected to preliminary identification tests and results are shown in Table 3. Ten of the isolates were Gram negative rods while nine were Gram negative cocci. In addition, seven of the Gram negative rods shaped bacteria were able to ferment the carbohydrates in the TSI medium. However, only two of these isolates produced hydrogen sulphide gas which is a strong characteristic of *Salmonella* strains. A total of nine rod shaped isolates were able to utilize citrate and only two of these produced gas.

**Table 3:** Preliminary identification test results for presumptive coliform bacteria isolates with m-FC agar (+ = positive for the test; − = negative for the test).

Isolate ID	Gram Staining	TSI				Citrate Utilization		
		Butt	Slant	Gas	H_2_S	Butt	Slant	Gas
A_1_	− (rod)	+	+	+	−	−	+	+
A_2_	− (rod)	+	+	−	−	+	+	−
A_3_	− (coccus)	+	+	−	−	−	+	−
B_1_	− (rod)	+	+	−	−	−	+	+
B_2_	− (rod)	+	−	−	+	−	+	−
B_3_	− (rod)	+	+	+	−	−	+	−
C_1_	− (coccus)	+	−	−	−	+	+	−
C_2_	− (rod)	+	−	−	−	−	+	−
C_3_	− (rod)	+	−	−	−	+	+	−
D_1_	+ (coccus)	−	−	−	−	−	+	−
D_2_	− (coccus)	−	−	−	−	+	+	−
D_3_	+ (coccus)	+	−	−	+	+	+	−
E_1_	− (coccus)	+	−	−	−	−	+	−
E_2_	− (coccus)	−	−	−	−	+	+	−
E_3_	− (coccus)	+	−	−	+	−	+	−
F_1_	− (rod)	+	+	−	−	−	+	−
F_2_	− (rod)	+	+	−	−	−	+	−
F_3_	− (rod)	+	+	−	+	−	−	−
G_1_	− (coccus)	+	−	−	−	+	+	−
G_2_	− (coccus)	+	−	−	−	+	+	−
G_3_	− (coccus)	+	+	−	−	−	+	−

The isolates from m-ENDO agar were subjected to preliminary identification tests and results are shown in Table 4. Seven of the isolates were Gram negative rods while three were Gram positive rods. Eight of the Gram negative rod shaped isolates partially utilized carbohydrates in the TSI agar but none produced gas. However, only one of these isolates produced hydrogen sulphide gas. All the isolates from m-ENDO agar did not produce gas from the Simmon’s citrate agar and only four were able to completely utilize citrate.

**Table 4:** Preliminary identification test results for presumptive coliform bacteria isolates with m-ENDO agar (+ = positive for the test; − = negative for the test).

Isolate ID	Gram Staining	TSI				Citrate Utilization		
		Butt	Slant	Gas	H_2_S	Butt	Slant	Gas
A_1_	− (rod)	−	−	−	+	−	+	−
A_2_	− (rod)	+	−	−	−	−	+	−
A_3_	− (coccus)	+	−	−	−	+	+	−
B_1_	− (rod)	+	−	−	−	−	+	−
B_2_	− (coccus)	+	+	−	−	+	+	−
B_3_	− (coccus)	+	−	−	−	−	+	−
C_1_	− (coccus)	+	−	−	−	−	+	−
C_2_	− (rod)	+	−	−	−	+	+	−
C_3_	− (rod)	+	−	−	−	+	+	−
D_1_	+ (coccus)	+	−	−	−	+	+	−
D_2_	− (coccus)	−	−	−	+	−	+	−
D_3_	− (rod)	+	+	−	−	+	+	−
E_1_	− (rod)	+	−	−	−	−	+	−
E_2_	− (coccus)	+	−	−	−	−	+	−
E_3_	− (coccus)	−	−	−	+	+	+	−
F_1_	− (coccus)	+	+	−	+	+	+	−
F_2_	+ (rod)	+	+	−	−	+	+	−
F_3_	− (coccus)	+	+	−	−	−	+	−
G_1_	− (coccus)	+	−	+	−	+	+	−
G_2_	+ (rod)	−	−	−	−	+	+	−
G_3_	+ (rod)	+	−	−	+	+	+	−

A number of morphological and biochemical parameters have been used to facilitate in determining the identities of faecal contaminating bacteria in water [25,26]. Despite the fact that the sensitivity of these protocols might not be very reproducible between laboratories, it is highly recommended that they should be combined with confirmatory biochemical tests.

The identities of the isolates from both m-FC and m-ENDO were confirmed based on their biochemical profiles and results are shown in Table 5. Amongst the *Enterobacteriaceae*, *Escherichia coli* were most frequently isolated from m-FC agar (7/21) and m-ENDO (8/21) respectively. In addition, two isolates from m-FC agar were positively identified as *Salmonella* species while only one isolate was confirmed as *Klebsiella* specie. Similar findings were observed from m-ENDO agar. Some of the isolates produced unknown profiles.

The detection of *Escherichia coli*, *Salmonella* species and *Klebsiella* species (Table 5) in borehole water that is intended for human consumption was a cause for concern. These isolates may pose severe health complications to humans especially if they harbour virulence gene determinants. These *E. coli* strains may belong to recently identified pathogenic serotypes such as *E. coli* O157:H7 and *E. coli* O104:H4 that have been reported to cause diseases in humans [27]. It has been established that domestic and wildlife animals are the natural reservoirs of bacteria belonging to the family *Enterobacteriaceae* and the presence of these bacteria in the environment results through the uncontrolled release of faeces [28]. During sample collection it was observed that some of the boreholes have been constructed next to pit latrines and this has the potential of contaminating groundwater [29].

**Table 5:** Identities of the isolates from m-FC and m-ENDO based on the biochemical profiles.

Isolate ID	m-FC	m-ENDO
A_1_	*Escherichia coli*	*Salmonella* species
A_2_	*Escherichia coli*	*Escherichia coli*
A_3_	Unknown profile	Unknown profile
B_1_	*Escherichia coli*	*Escherichia coli*
B_2_	*Escherichia coli*	Unknown profile
B_3_	*Salmonella* species	Unknown profile
C_1_	Unknown profile	*Escherichia coli*
C_2_	*Escherichia coli*	*Escherichia coli*
C_3_	*Escherichia coli*	*Escherichia coli*
D_1_	Unknown profile	Unknown profile
D_2_	Unknown profile	Unknown profile
D_3_	Unknown profile	*Escherichia coli*
E_1_	Unknown profile	*Klebsiella* species
E_2_	Unknown profile	Unknown profile
E_3_	Unknown profile	Unknown profile
F_1_	*Klebsiella* species	Unknown profile
F_2_	*Escherichia coli*	*Escherichia coli*
F_3_	*Salmonella* species	Unknown profile
G_1_	Unknown profile	Unknown profile
G_2_	Unknown profile	*Escherichia coli*
G_3_	Unknown profile	*Salmonella* species

## 4. Conclusions

The study has revealed that borehole water of Magogoe village is vulnerable to physico-chemical as well as bacteriological pollution. It was found that change in the seasons (from winter to spring) did not have any impact on the quality of water except for the microbial quality of the borehole water which deteriorated significantly during spring. Therefore, groundwater may not always be of pristine quality as is perceived.

For this reason, it is recommended that groundwater for human consumption is treated in the same manner as surface water sources before distribution to users. Detailed and continuous monitoring and assessment of other chemical species in the area such as total phosphorus concentrations which are indicative of pollution from human and animal waste is highly recommended. Increasing the frequency of sampling and analysis is also needed to effectively monitor the quality of the borehole water. Early detection of possible contamination can lead to faster implementation of corrective measures, preventing an imminent waterborne disease outbreak.

Communities using borehole water as their source of water should be educated of the possible risks when borehole water is used for human consumption. Education should also include possible means of treatment of water such as boiling and use of chlorination tablets so as to prevent possible adverse health effects. In addition, community participation through protection of drinking water sources from contamination could help improve the water situation in the area thereby ensuring a health environment. For example, regulations governing activities in the area especially pit latrine siting, best management practices for agriculture, general hygiene and appropriate storage practices at household level.
